# Analysis of the Erosivity of High-Pressure Pulsating Water Jets Produced in the Self-Excited Drill Head

**DOI:** 10.3390/ma14154165

**Published:** 2021-07-27

**Authors:** Monika Szada-Borzyszkowska, Wojciech Kacalak, Dariusz Lipiński, Błażej Bałasz

**Affiliations:** Faculty of Mechanical Engineering, Koszalin University of Technology, Racławicka 15-17, 75-620 Koszalin, Poland; monika.szada-borzyszkowska@tu.koszalin.pl (M.S.-B.); wojciech.kacalak@tu.koszalin.pl (W.K.); blazej.balasz@tu.koszalin.pl (B.B.)

**Keywords:** water jet, pulsed water jet, FEM, simulations, self-excited pulsating head, hydro jetting-erosion

## Abstract

The dynamic impact of a water jet with a periodically changing structure can be used in various industries. The paper presents a design solution for a self-excited pulse head. This head can be used in mining for drilling holes and breaking rocks. The design of the head was developed based on computer simulations, which made it possible to learn the mechanism of impulse shaping inside the head. Tests of the water jet produced in the self-excited pulsation head showed the occurrence of periodic changes in its internal structure and pulsation frequency. A significant increase in the dynamic stream pressures was demonstrated for the head working in the water environment compared to the head working in the air environment For example, for nominal medium and highest pressures, this increase is up to 82%, while for the lowest pressures (10 MPa), the pressure force values increase by 46%. It was found that an increase in the nominal water pressure causes a decrease in the frequency of hydrodynamic pulses in the head operating in both the water and air environment.

## 1. Introduction

The use of pulsating water jets in mining is a new issue. A pulsating water jet can be used to intensify rock breaking and in the production of oil and gas. There are many varieties of hole drilling technology using a high-pressure water jet. Among them are the methods using a pulsating water stream.

The impulse water jet is used, among others, in drilling technology. Various designs of nozzles shaping a pulsating water stream are known. The results in [1] show that pulsating flows can significantly increase the speed of drilling an oil well. There is still a search for better solutions for the use of a pulsed jet in the drilling technology in oil mining [2,3]. A pulse head with specific geometrical parameters of the vortex chamber is usually used to generate such a stream. The advantage of such heads is the lack of moving parts. The most important technical problems include the strong dependence of the stream properties on the design parameters of the self-excited pulse heads.

A pulsed jet of high intensity and powerful water can potentially also be an effective and alternative tool for secondary rock breaking. Effective crushing of rocks by the pulsating stream of water ensures the intensification of the rock cracking energy with a reduced reaction force on the handle of the working device. Rock fracture is also affected by the distance of the water jet from the processed material and the structural structure of the processed material. In the work of Dehkhod [4], the influence of the pulsating stream on the rock fracture process was investigated. The research showed the formation of deep internal damage in the layers of the tested material.

The innovative generation of a pulsating water jet was developed by the Vijay team [5]. It consists in generating hydrodynamic pulses formed using a device that segments a water stream with an ultrasonic frequency. Works on the further development of this method were also carried out by Foldyna’s team [6]. The use of tubular nozzles for creating waves in a stream in this method was described by Nebeker and Rodriguez [7,8], and then developed by Chahine [9]. However, few of these solutions have found industrial applications.

Foldyna’s team is working on generating hydrodynamic pulses formed with the use of an ultrasonic frequency segmentation device [10]. In their work, they develop models for the visualization of the water stream structure and the analysis of the flow velocity field. Knowledge of the geometry and velocity fields of pulsating water jets generated by the high-pressure system is important for the optimal selection of parameters in terms of machining efficiency. Flow visualization methods significantly contribute to the improvement of new pulse systems at the design stage.

Theoretical analyses concerning the generation of pulsations in water streams are included in the works of Heymann [11] and Hung [12]. They describe the phenomena of the accumulation of cyclic high-frequency, hydrodynamic interactions of the stream energy on the treated surfaces [6]. It has been shown that the maximum pressure of a pulsating stream, may several times exceed the pressure in the field of a continuous stream. This was also confirmed in the experiments of Smith and Kinslow [13].

The properties of the high-pressure water stream are determined by the method and conditions of the stream shaping and its features, such as velocity distribution of fluid particles and uniformity of mass distribution [14,15]. The effectiveness of the machining process is assessed based on stream coherence (stream hydrodynamic properties). The ability of the water stream to not disperse during movement and to maintain its energy potential is mentioned as the main condition for the efficiency of the process [16].

The high-pressure water jet at the outlet of the nozzle hits the air, i.e., a medium with a density approximately 800 times lower than that of water [17]. With the influence of the medium and turbulent pulsations [18,19], as a result of mass and energy exchange [20], the stream slightly expands. It also undergoes aeration and a certain change of its original shape takes place [17]. In the initial high-pressure zone of the stream, there is a compact core [21] with an almost constant speed and pressure, which changes in the transition zone. In order to increase the erosivity of the high-pressure water jet, various types of abrasives [22,23,24] are added, which has been demonstrated as a result of many research works [25,26].

The complexity of the process of pulsating flows is influenced by such factors as the size and shape of the stream, velocity distribution in the cross-section, surface tension of the liquid, flow turbulence, the influence of the immediate environment surrounding the stream [27]. In the work of Nebeker [8], the phenomena of the pulsating streamflow were described, thanks to the tests with the use of a rotating head with holes on the perimeter, ensuring the modulation of the water outflow speed as a function of time.

The advantages of using a discontinuous water jet with a pressure of 65 MPa with the modulation frequency of pulses 2 kHz and 5 kHz and the use of a nozzle with a diameter of 1.5 mm are illustrated with the test results [28] of the rock erosion process. They showed a fourfold increase in limestone eroding efficiency and a correspondingly twofold increase for sandstones.

Wyli’s analyses [29] also confirm that the use of a sinusoidally modulated water jet entails an increase in power by only 18% compared to the non-modulated jet. Nevertheless, more than a twofold increase in the output of rock materials is ensured, while the use of a water jet with a pressure modulated according to a rectangular cycle provides a more than three-fold increase in such output.

The discontinuity of the water stream structure can be obtained by various methods. One of them is the generation of hydraulic pulses at the outlet of a specially designed pulse head. The aim of the article is to conduct computer simulation and experimental tests with the use of a self-excited pulse head. In simulation tests, favorable head design solutions were determined. Then, tests were carried out on the selected design solution of the self-excited pulse head. The influence of the construction parameters of the self-excited pulsation head on the stream velocity generated in the outlet nozzle was determined.

## 2. Methodology of Simulating Pulsating Flows

The mechanism of pulse variability in the water stream flowing through such a head is a complex phenomenon [30]. The formation of hydrodynamic impulses occurs as a result of negative pressure, which is generated in specific zones of the chamber, which favors the formation of vortices. The resulting vortices also contribute to the pulse modulation of the water stream and the formation of sudden pressure increases [3], which results in high rock drilling efficiency [30,31]. Moreover, the pulse head periodically causes a negative pressure in the drilling zone, which facilitates the removal of eroded rock particles [32].

**Figure 1:**
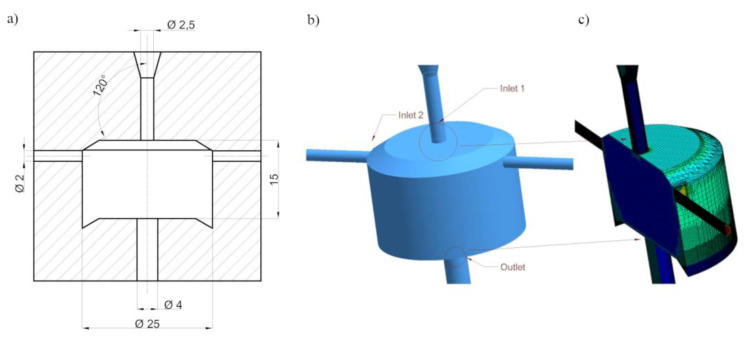
A simplified physical model of the pulse head: a scheme of the construction (**a**), computer model (**b**), model after meshing (**c**).

Computer simulations were necessary to analyse the stream flow through the self-excited pulsed head and to determine the geometric features of the head (Figure 1). Their goal was to determine the beneficial design solutions of the head. Then, tests of the selected design solution of the self-excited pulse head were carried out. The influence of the construction parameters of the self-excited pulsation head on the stream velocity generated in the outlet nozzle was determined.

The autors determined relations between the design parameters of the head and the shape and erosive efficiency of the generated water stream with the use of SolidWorks Flow Simulation software (Dassault Systèmes, Vélizy-Villacoublay, France)

The following assumptions were made for simulation studies using the Solid Works Flow Simulation software:

- numerical calculations of turbulence were performed using the k−ε model and its standard coefficients,
- the calculations were made with the use of a structural mesh in the boundary area (y+ < 1) and a tetragonal mesh outside the boundary layer,
- the structural mesh near the wall has been compacted and consists of 3 layers,
- in total, the grid contains 563 thousand elements (further increasing the number of elements did not change the results of numerical calculations—velocity distribution in the nozzle outlet profile),
- the simulation process ended automatically after reaching the convergence criterion, which required the execution of at least 500 iterations,
- the simulation was carried out in the steady-state, because the determination of the existence of vortices in the head’s swirl chamber, and not the very method of their formation, determined the proper operation of the head,
- wall roughness is omitted,
- flow through the head was forced by the pressure defined in the inlet nozzle,
- side channels and the outlet nozzle are defined by the pressure corresponding to the value of the ambient pressure,
- water during the simulation was treated as an incompressible fluid which is a continuous phase,
- there is no external heat exchange, the fluid temperature is constant [3].

The used k−ε model is characterized by low sensitivity to inlet conditions for the quantities describing turbulence. This feature is important because these values are not exactly known for different design solutions. The k−ω model more fully models the turbulent flow in the boundary layer, but is very sensitive to the free flow features. The k−ω shear stress transport (SST) model is a model that combines the advantages of the k−ε model and the k−ω model and includes a term limiting the value of the kinetic energy of turbulence in the areas of strong (positive) pressure gradients.

The authors analyzed the results of combining the positive features of both models by transforming the k−ε model into the equations for k and ω. This transformation takes into account that ω is the proper (unit) dissipation of the kinetic energy of turbulence, i.e., ω = ε/k. The equations of the k−ε model were multiplied by a function that has a value of 1 in free flow and zero in the boundary layer. The need to apply the dependence limiting the value of the main stresses of the flow— shear stress transport (SST) was taken into account. This model is suitable for describing 3D flows with strong changes in flow direction and strong rotations. Rather, it is useful for well-defined inlet conditions.

## 3. Simulation Studies of the Influence of the Side Holes Location

**Figure 2:**
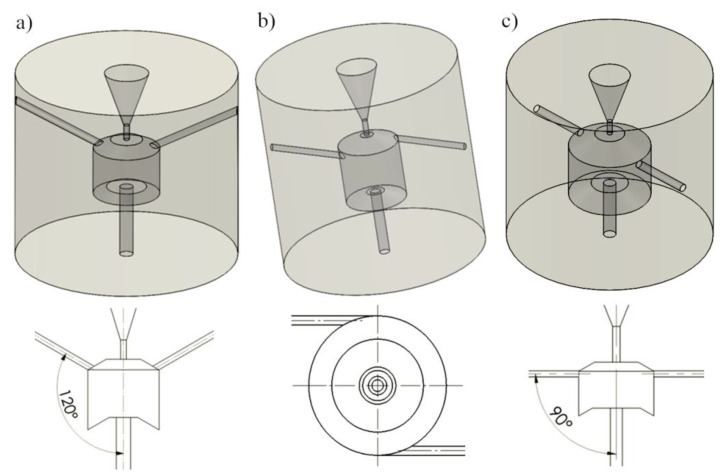
Differentiation of the geometry of the self-excited pulse head with different locations of the side holes: (**a**) inclined at an angle of 120° to the axis of the head; (**b**) tangential to the swirl chamber; (**c**) perpendicular to the axis of the head.

In the simulation tests, a maximum diameter of the inlet nozzle of 2.5 mm was assumed for an output of 160 dm^3^/min and a nominal pressure of 150 MPa. These studies aimed to analyse velocity and pressure vector distributions in terms of the possibility of creating vortex rings. The speed of the water stream at the outlet from the self-excited pulse head depended on the characteristics of the circular vortices. In the first stage, computer simulation tests were carried out for different positions of the side openings relative to the axis of the swirl chamber. Figure 2 shows various geometric variants of the self-excited pulsed head, developed for computer simulation studies of the water stream flow through the head.

Three design solutions were analysed, in which the side openings were located: at an angle of 120° to the head axis (Figure 2a), tangent to the swirl chamber (Figure 2b), and perpendicular to the swirl chamber (Figure 2c). For each head design, the pressure and velocity vectors distributions inside the self-excited pulse head were determined. The parameters tested and their scope in a full factorial experiment are presented in Table 1.

**Figure 3:**
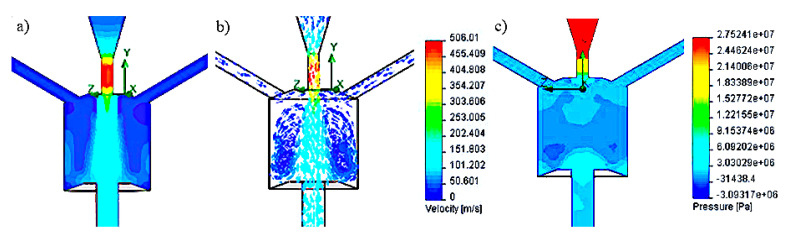
Distribution test results: (**a**,**b**) speed and (**c**) pressure inside the self-excited pulse head with side openings located at an angle of 120° (H = 20 mm, D = 20 mm, d_2_ = 3 mm, d_3_ = 5 mm, λ = 110°, h_1_ = 8 mm), for a nominal pressure of 150 MPa.

The simulation results of velocity and pressure vectors for the head with side openings at an angle of 120° to the swirl chamber axis are shown in Figure 3.

These results were obtained for the following geometric parameters: H = 20 mm; D = 20 mm; d_2_ = 3 mm; d_3_ = 5 mm; λ = 110°; h_1_ = 8 mm; the inlet pressure of 150 MPa, the maximum speed at the head outlet was 506 m/s, while the average outflow velocity was 94 m/s. It has been shown that for the nominal water pressure of 150 MPa, the velocity and pressure distributions, as well as the shape of the vortex rings inside the chamber, do not favorably influence the hydraulic impulses. The reason is the outflow of the water stream through the side holes. On the other hand, in the conditions that ensure that the working medium is sucked through the side openings, the water stream flowing from the head does not reach a sufficiently high speed.

**Figure 4:**
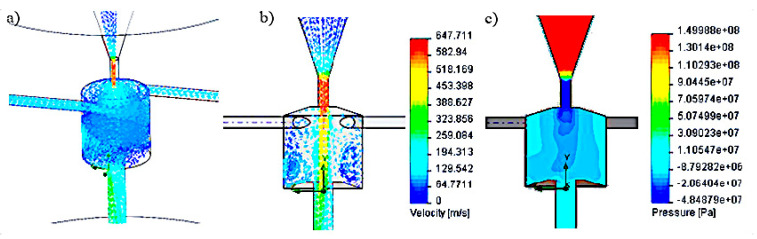
Distribution test results: (**a**,**b**) speed and (**c**) pressure inside the self-excited pulse head with side openings tangent to the swirl chamber.

Subsequent simulation tests were carried out for side holes located tangent to the swirl chamber. Examples of test results for the head with such side openings, at a nominal water pressure of 150 MPa, are illustrated in Figure 4.

The above head model is characterized by the following swirl chamber parameters: H = 20 mm; D = 20 mm, d_2_ = 3 mm, d_3_ = 5 mm, λ = 110°, h_1_ = 8 mm. Simulation tests carried out for the tangential location of the side holes relative to the swirl chamber, for a pressure of 150 MPa, showed at the exit of the head the maximum speed of the water stream of 647 m/s and the average speed of 315 m/s. The analysis of the results of research on the velocity and pressure vectors distributions in the pulse head with side openings located tangentially shows the variable shape of the vortex rings, which disturb the mechanism of generating water pulses.

**Figure 5:**
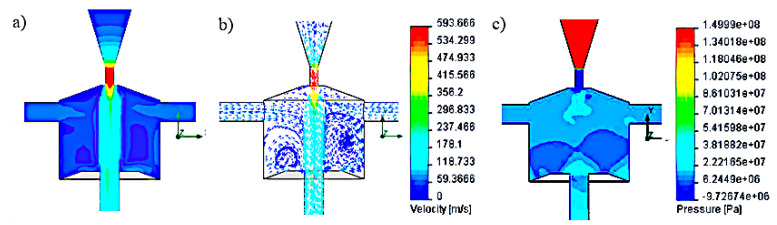
Distribution test results: (**a**,**b**) speed and (**c**) pressure inside the self-excited pulse head with side openings perpendicular to the swirl chamber.

In the next stage, computer simulations were carried out for the pulse head with side holes perpendicular to the axis of the vortex chamber. The simulation tests were carried out for various models of self-excited pulse heads. One of the examples of simulation results of velocity and pressure distributions occurring in this type of head is shown in Figure 5.

The results of computer simulations were obtained for the head with side openings perpendicular to the swirl chamber with the following geometry: H = 25 mm; D = 32 mm; d_2_ = 6 mm; d_3_ = 6 mm; λ = 110°; h_1_ = 6 mm. The maximum speed of the water stream flowing into the vortex chamber of such a head at a nominal water pressure of 150 MPa was 593 m/s, and its average velocity at the outlet of this head was 245 m/s. The analysis of the results of velocity and pressure distribution inside the self-excited pulsation head with side openings perpendicular to the axis of the vortex chamber showed that the advantageous shape of the vortex rings and pressure distribution is obtained. This has a positive effect on the formation of stable water pulses.

Among the three most advantageous variants of the pulse head analyzed (Figure 2), in the model of the location of the side holes at an angle of 120° inside the swirl chamber (Figure 3), similar values of the water jet velocity are observed. Such a situation, despite the existence of clearly differentiated pressure gradients, does not favor the formation of vortex movements.

In the case of the head with tangential side openings to the vortex chamber (Figure 4), there are clear swirls of individual water layers in it. Additionally, rotation of the water flow with respect to the axis of the vortex chamber occurs. As a result, a similar pressure is present in the entire volume of the swirl chamber, which causes the water to flow outwards also through the side openings, preventing the formation of vortex rings. This prevents the initiation of pressure pulses in the core of the water stream flowing along the axis of such a head.

The most favorable situation appears in the model of a self-excited pulse head with side openings perpendicular to the vortex chamber (Figure 5), for which the distribution of water ring swirls and the pressure distribution at the outlet of this chamber favor the generation of pressure pulses in the flowing water stream.

The criterion for assessing many variants of the position of the side openings axes was the formation of vortex displacements in planes passing through the axis of the swirl chamber, the absence of vortices around the chamber axis, and the possibility of creating a negative pressure at the inlet to the chamber. For the system with inlet openings perpendicular to the axis of the swirl chamber, many variants of the pulse head have been developed, of which the three most favorable are presented in Table 2.

**Figure 6:**
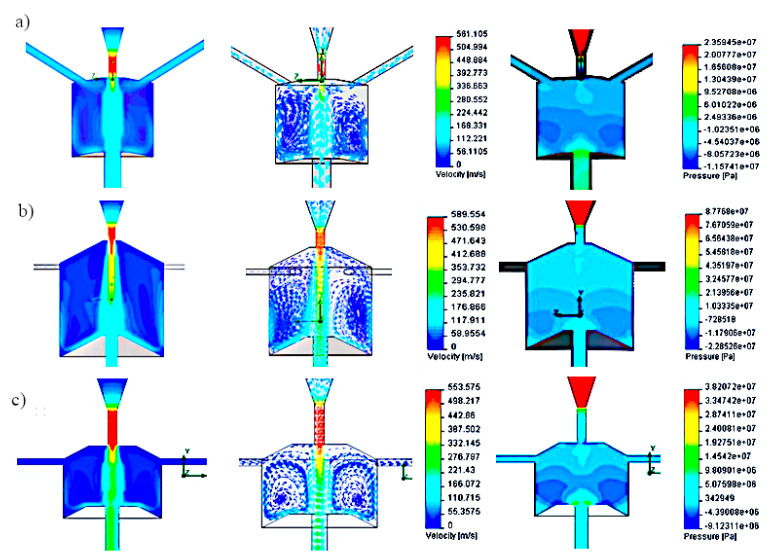
Graphical interpretation of velocity and pressure distributions for selected head solutions with different locations of side openings: (**a**) inclined at an angle of 120° to the axis of the head; (**b**) tangential to the swirl chamber; (**c**) perpendicular to the axis of the head.

Also for the remaining solutions, various design variants were assessed and the most favorable of them for heads with different locations of side openings are shown in Figure 6.

For the variant of the pulse head with side openings at an angle of 120° to the axis of the head, a dense filling of the interior of the swirl chamber with quite uniform velocity vectors is achieved, which does not favor the formation of vortex movements. In the case of a head with tangential side openings relative to the axis of the swirl chamber, there are distinct swirls of individual water layers. However, there is rotation in relation to the axis of the vortex chamber. As a result, the pressure is equalized throughout the entire volume of the swirl chamber. This causes the water to run out also through the side openings. This hinders the formation of vortex rings and significantly reduces the initiation of pressure pulses in the core of the water stream flowing along the axis of such a head. The most favorable conditions are found in a self-excited pulse head with side openings perpendicular to the vortex chamber. Clear swirls of water rings and pressure increase at the outlet from this chamber are observed, which favors the generation of pressure pulses in the outgoing water stream.

The comparison of the results of simulation tests of the best variants for each type of self-excited pulsation head is presented in Table 3. It contains geometrical parameters of such heads, the location of side holes, and data on the water jet velocity at the inlet and outlet of these heads.

The data contained in this table shows that the highest outlet velocity of the water jet is provided by the self-excited pulse head with side openings tangent to the vortex chamber. Only 5% lower speed is achieved by the water stream flowing from the head having side holes perpendicular to the swirl chamber. The speed of the water stream produced in the pulse head with side openings situated at an angle of 120° to its axis is as much as 2.5 times lower. Therefore, taking into account the relationships of the above-mentioned velocities of the water stream, as well as the most favorable distributions of velocity and pressure vectors inside the vortex chamber (Figure 6), it can be clearly stated that the best solution for a self-excited pulse head is a head with side holes perpendicular to the vortex chamber.

## 4. Research on the Model of the Pulsating Head Intended for Drilling Holes

The search for the best geometric parameters of the self-excited pulse head was carried out using the Solid Works Flow Simulation software. These tests aimed to search for an optimal model of the pulsation head with regard to the maximization of the outlet velocity of the water stream, taking into account the suction of the external medium through the side holes and appropriate shaping of the vortex rings and the effectiveness of using the head at water pressures from 15 MPa to 150 MPa. For this purpose, further tests of the self-excited pulse head were carried out for the geometric parameters presented in Table 4.

Based on the test results, the dimensional parameters of the self-excited pulsation head model, intended for drilling holes with a water jet in the pressure range of 15 ÷ 150 MPa, were determined. The optimal geometrical dimensions of the head selected based on the conducted research and analyses are presented in Table 5.

**Figure 7:**
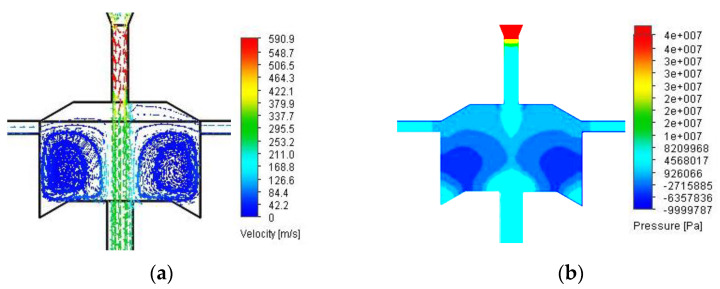
Distribution of velocity vectors: (**a**) and pressure (**b**) inside the selected model of the self-excited pulse head at a nominal pressure of 150 MPa.

The distributions of velocity and pressure vectors inside of the self-excited pulse head are shown in Figure 7.

The analysis of velocity vector distributions indicates the presence of symmetrical hydraulic vortices almost in the entire volume of the vortex chamber, which, with the suction of the external medium and the zone-symmetric pressure distribution in this chamber, provides conditions for the formation of hydrodynamic pressure pulses in the core of the water stream flowing through the self-excited head pulsating.

## 5. Methodology of the Experimental Research

To verify the effectiveness of the pulse head operation in water and air environment, appropriate experimental studies were carried out. This required the construction of test stands and their equipment with appropriate technological and specialized measuring apparatus. The constructed test stands provided the possibility of measuring physical quantities and technological indicators characterizing the operational properties of the head. The water monitor was the source of the water at the specified pressure and flow rate. To produce a high-pressure impulse water stream, in addition to the necessary equipment, appropriate designs of self-excited pulsation heads were used. To carry out measurements of selected physical quantities and indicators characterizing the impulse structure of the water stream, it was required to assemble specialized apparatus and measuring instruments.

### 5.1. Test Stand for the Head Working in the Air Medium

**Figure 8:**
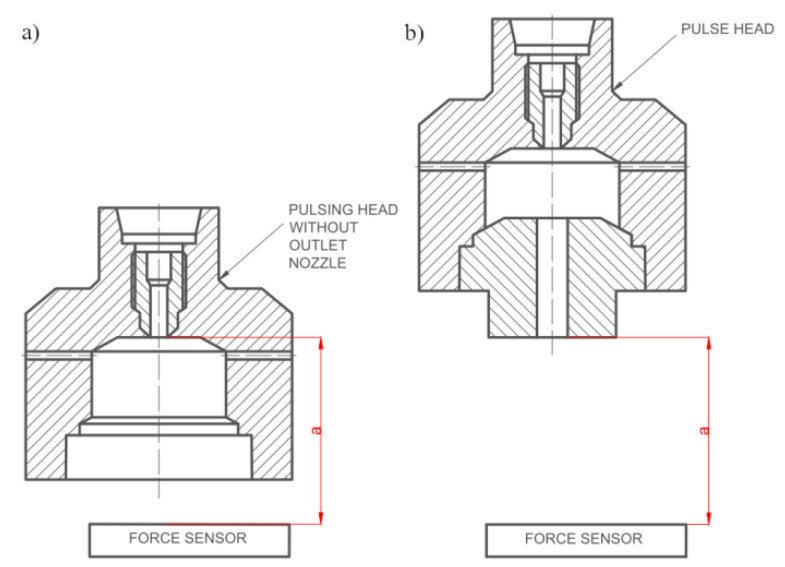
Graphical interpretation of the distance between the pulse head and the force sensor: (**a**) for a continuous stream, (**b**) for a pulsed stream.

In the test stand for the self-excited pulse head working in the air medium, a piezoelectric force sensor and an ultrafast camera (Phanton V12.1, Vision Research, Wayne, NJ, US; which enables the recording of images at a speed of 1,000,000 frames per second, with a resolution of 1280 × 800 pixels and a sensitivity of 6400 ISO/ASA) were used to measure the frequency of the water jet pulses. Tests in the air environment were carried out for specific distances between the head and the piezoelectric force sensor (frequency range: 0–10 kHz; linearity %FSO (full scale output) ≤ ±1.5; hysteresis %FSO ≤ 1.5). Figure 8 shows the graphical interpretation of the distance measurement for pulsed and continuous jets.

The measurement of the frequency of the generated pulses was as follows: from the P30 monitor, a water jet of appropriate pressure was fed through a high-pressure hose to the entrance to the self-excited pulse head mounted in a special holder. Underneath, a force sensor was mounted, which, using a piezoelectric transducer, registered the variability of the water jet pressure and transmitted the generated signal to the computer.

**Figure 9:**
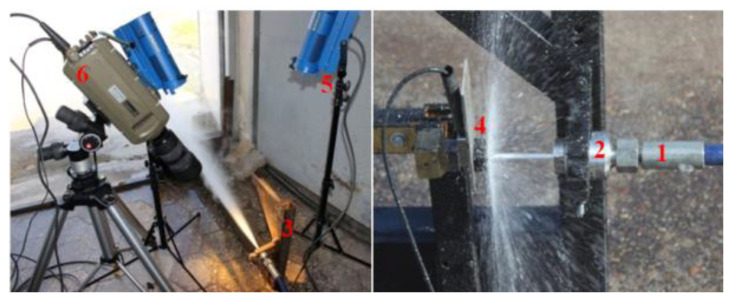
Measuring stations for recording images of impulse and continuous water jets operating in the air medium: 1−high-pressure power cord, 2−self-excited pulse head, 3−head mounting bracket, 4−force sensor, 5−lighting, 6−Phantom V12.1 camera.

To compare the frequency of the generated pulses recorded by the force sensor, images of the pulsating water stream were also recorded with the use of the ultra-fast Phantom V12.1 camera. Correct illumination of the measurement zone was ensured by the use of VR-L2400 reflectors (Pallite, München, Germany) which illuminate the water stream at the exit of the head. The impulse water stream operating in the air medium was registered with 11 000 frames per second (Figure 9).

The frequency of water pulses generated in the self-excited pulse head in the air environment was registered as follows. A water jet of appropriate pressure, produced in a hydromonitor, was fed via a high-pressure conduit to the entrance to a self-excited pulsed head, in which an impulse water jet was generated. The pulsing jet coming out of the pulse head affected the piezoelectric force sensor, and its image was recorded by an ultra-fast camera.

### 5.2. Test Stand for the Head Working in the Water Medium

**Figure 10:**
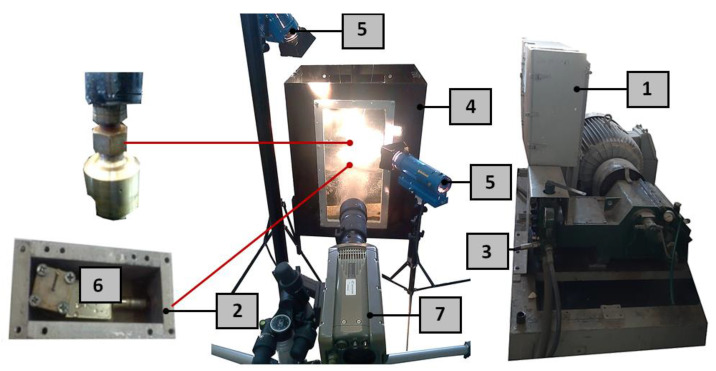
Measuring station for testing the water stream produced in a self-excited pulse head working in an aqueous medium: 1−P30 monitor, 2−force sensor mounting, 3−high-pressure hose, 4−water tank, 5−illuminator, 6−force sensor with a KISTLER 9602AQ01 transducer, 7−Phantom V12.1 camera.

A test stand was built for laboratory tests of the head intended for drilling deep holes in the water medium, the general view of which is shown in Figure 10.

To carry out research in an aqueous medium, the pulse head was placed in a special holder. A force sensor with a piezoelectric transducer of the KISTLER-9602AQ01 type (Kistler, Winterthur, Switzerland), which was placed directly under the pulse head, was used to record the value of the thrust force of the flowing stream. It recorded the values of dynamic changes in the thrust force of the tested pulsed jet, transmitting the data to the computer. To compare the recordings of the frequency recorded with the force sensor, images of the stream were additionally recorded using an ultrafast Phantom V12.1 type TV camera. The course of research on the operation of the pulsating head for surface treatment was as follows: water of appropriate pressure, produced in the hydromonitor, was sent using a high-pressure hose directly to the entrance to the pulsating head. The individual phases of the process of shaping the pulses in the water stream at the output of the self-excited pulse head were recorded using a camera recording 4000 frames per second and transmitted to a computer. The record of dynamic changes in the thrust force of the impulse stream generated in the pulse head was recorded with a force sensor and transferred to a computer where these records were analysed.

### 5.3. Method of Assessing the Erosiveness of the Impulse Stream

**Figure 11:**
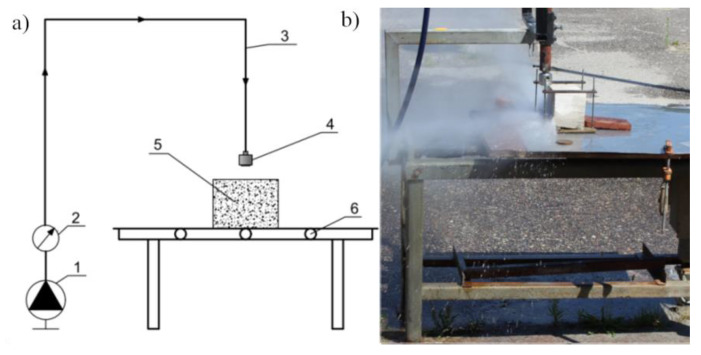
Stand for testing water jet erosivity: (**a**) stand diagram: 1−monitor unit, 2−manometer, 3−power cord, 4−self-excited pulse head, 5−workpiece, 6−sliding table, and (**b**) general view of the stand.

A sliding table on which the processed material was placed was used to test the erosivity of the pulsed stream produced in the self-excited pulsation head intended for surface treatment. The view of the stand is presented in Figure 11.

The table movement was carried out employing a ball-screw gear driven by a DC electric motor with various feed speeds ranging from 3 to 20 mm/s. The method of mounting on the head position enables adjustment of its distance from the processed material in the range of 0 ÷ 150 mm.

## 6. Results and Discussion

### 6.1. Research on the Characteristics of the Water Stream Produced in a Self-Excited Pulsed Head Intended for Holes Drilling

The variant of the self-excited pulse head for drilling holes was determined based on simulation tests. The appropriately selected geometry of this head, especially the shape of its swirl chamber and the system of side openings, make the distinct water swirls arising in this chamber intensively modulate the centrally flowing water stream, causing periodically changing pulsations in it. To assess the suitability of such a pulsating head intended for drilling holes, tests were carried out on the characteristics of the water jet, assessing its structure. For this purpose, an ultrafast TV camera of the Phantom V12.1 type was used, which recorded the heterogeneity of the structure of the pulsating water stream. Such a drilling head was tested at the following nominal pressures of the water jet: 10, 15, 20, 25 MPa

**Figure 12:**
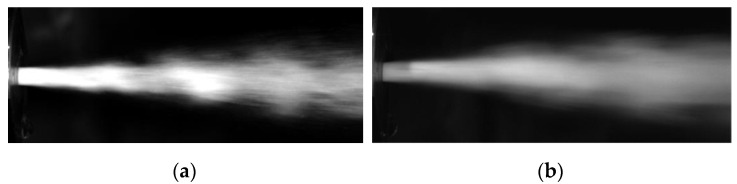
Images of the formation of hydrodynamic pulses during the operation of the head in the air medium, working at the nominal pressure: (**a**) 10 MPa, (**b**) 25 MPa.

Examples of recorded images of the formation of hydrodynamic pulses for the head working in the air environment are presented in Figure 12. The obtained images recorded with an ultrafast TV camera during the operation of the head in the air medium at different input pressures indicate a discontinuity of the water stream.

**Figure 13:**
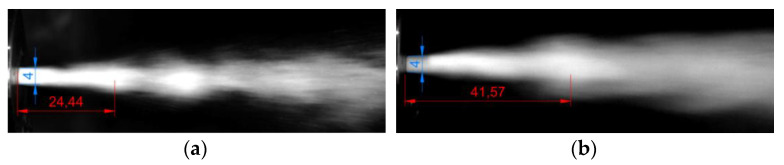
Sample images of a pulsating water stream flowing from the outlet nozzle, tested under air conditions, working at the nominal pressure: (**a**) 10 MPa, (**b**) 25 MPa.

Confirmation of the phenomenon of the water impulse formation required an assessment of the structure of the pulsating jet generated at the exit of the drill head. Examples of measurements of the distance at which the impulses are shaped from the outlet nozzle of the tested head are shown in Figure 13.

Measurements of the shape of the hydrodynamic pulses were made with the use of Auto-CAD software, which enabled scaling of the photos in relation to the characteristic dimension, i.e., the diameter of the outlet nozzle. Examples of such measurements are presented in Figure 13 and the results are summarized in Table 6.

**Figure 14:**
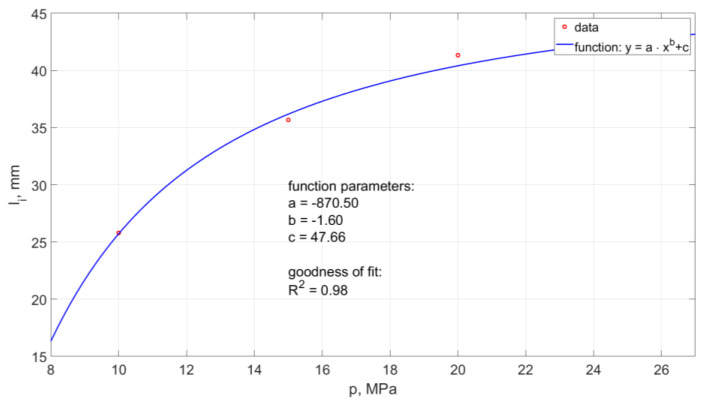
Influence of water pressure on the distance between the formation of hydrodynamic impulses and the outlet from a self-excited pulse head operating in the air environment.

The average distance of the generated hydrodynamic impulses from the outlet nozzle from such a head operating in the air environment is in the range of 24 ÷ 43 mm. The summary of the average values of the distance of shaping the pulses depending on the pressure is shown in Figure 14.

The model of this relationship determined by the method of least squares takes the form:

(1) $$l_{i}=-870.5\cdot p^{-1.6}+47.66$$

The model determination coefficient is approximately $R^{2}=$ 0.98.

### 6.2. Shaping of Impulses in the Water Stream Operating in the Water Environment

**Figure 15:**
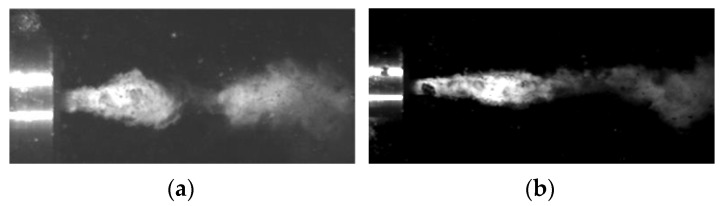
Results of the formation of hydrodynamic impulses in the water stream during the operation of the head in the water environment, at the input pressure: (**a**) 10 MPa, (**b**) 25 MPa.

The characteristics of the pulsating stream generated in the self-excited pulsation head intended for drilling holes were also tested for the head operating in the water environment. Examples of images of the heterogeneous structure of the water stream produced in the water environment, recorded with an ultrafast TV camera, are shown in Figure 15. The recorded images show the interrupted stream at the exit of the head (Figure 15a). Water pulses elongation with increasing nominal water pressure was observed (Figure 15b).

**Figure 16:**
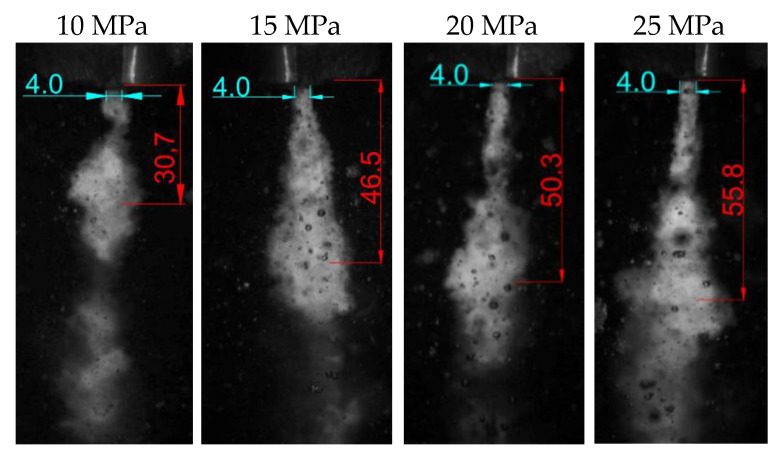
Examples of images of shaping hydrodynamic pulses produced in an aquatic environment.

Thanks to the recorded images, it was possible to determine the distance of the formation of hydrodynamic pulses from the outlet from the nozzle of the self-excited pulsed head for drilling holes (Figure 16). It was found that in the tested range, with increasing pressure, such pulses are shaped farther and farther away from the outlet nozzle. The summary of the average distance from the tested material for the head operating in the water environment is presented in Table 7.

**Figure 17:**
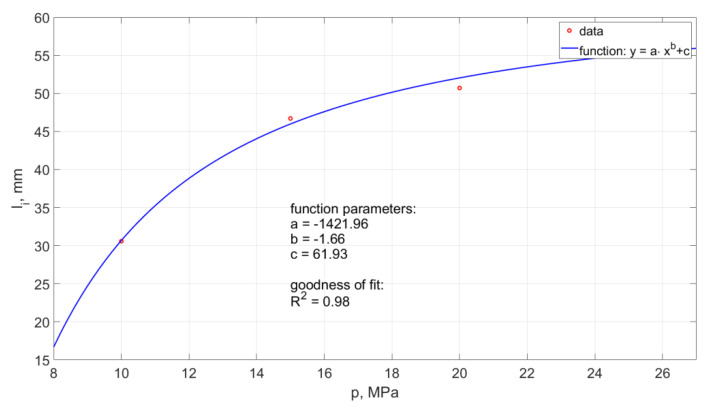
Influence of water pressure on the distance between the formation of hydrodynamic impulses and the outlet from a self-excited pulse head operating in the water environment.

The average distances of the hydrodynamic pulses from the outlet nozzle of the tested pulse head are presented in Figure 17.

The relationship takes the form:

(2) $$l_{i}=-1421.96\cdot p^{-1.66}+61.93.$$

The model determination coefficient is approximately $R^{2}=$ 0.98. Thus, in approximately 98%, the above model explains the variability of $l_{i}\left(p\right)$.

The obtained test results clearly show that with the increase in the pressure of the water stream, the “stretching” of the hydrodynamic pulses and the forming at an increasing distance from the outlet nozzle occurs.

### 6.3. Determination of the Frequency of the Formation of Water Pulses

Measurements of the frequency of occurrence of water pulses recorded at the outlet of the self-excited pulse head intended for drilling holes were made with the use of recorded images from an ultrafast TV camera of the Phantom V12.1 type. The images were then analysed in the Cine Viewer 675 program, which enabled the counting of the number of hydrodynamic pulses occurring in the analysed period.

#### 6.3.1. The Frequency of the Head Pulsation Stream in the Air Environment

**Figure 18:**
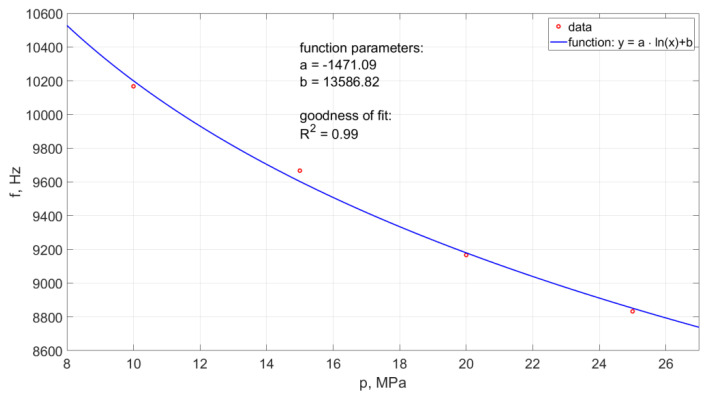
Influence of nominal water pressure on the frequency of pulses recorded with an ultrafast camera, for the head designed for drilling holes working in the air environment.

Determination of the frequency of pulsation pulses of the head operating in the air environment was made for the working pressure in the range: 10, 15, 20, 25 MPa. The results of measurements of the frequency of hydrodynamic impulses occurring in water streams for the given pressures are presented in Figure 18.

An increase in the nominal water pressure, increasing the outflow velocity of the stream generated in the self-excited pulse head, results in a reduction in the frequency of hydrodynamic impulses (Figure 18). This relationship is very precisely (R^2^ = 0.99) described by the following empirical Equation:

(3) f=−1471.09·ln(p)+13586.82

#### 6.3.2. Registration of the Frequency of the Head Pulsation Stream in the Water Environment

**Figure 19:**
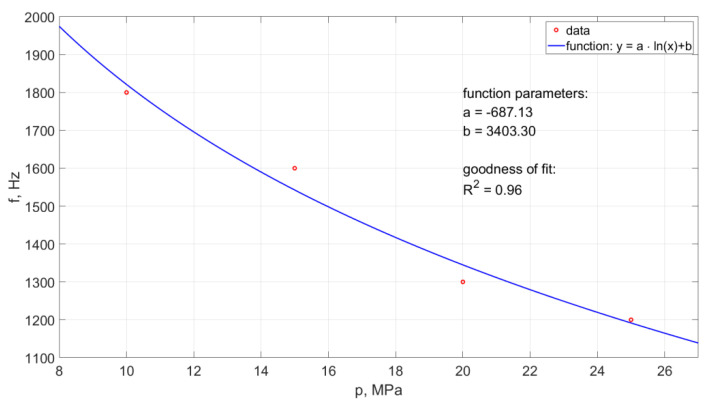
Influence of the change of nominal water pressure on the frequency of recorded hydrodynamic impulses, produced in a self-excited pulsed head designed for drilling holes, working in the water environment.

The frequency of the generated water pulses in the pulse drill head was also tested in the water environment. Examples of the results of such measurements made with the use of the ultrafast TV camera are presented in Figure 19. This diagram illustrates the effect of the nominal water pressure on the frequency of pulsations occurring in such operating conditions of the water stream.

In the case of the operation of the pulsating head in an aqueous environment, an increase in the nominal water pressure results in a reduction in the frequency of hydrodynamic pulses. Based on the collected data, the empirical relationship was determined:

(4) f=−687.13·ln(p)+3403.3

The model determination coefficient is approximately R^2^ = 0.96. Thus, in approximately 96% the above model explains the variability of f(p).

Based on this dependence, it is possible to determine the frequency of the formation of hydraulic impulses generated in the water stream flowing from such a self-excited pulse head for a given inlet water pressure in the considered range of physical conditions.

### 6.4. Determination of the Frequency of Occurrence of Water Pulses in a Pulsating Water Stream

The frequency of the occurrence of water pulses in the pulsating water stream, generated in the self-excited pulsed head intended for drilling holes, was also registered with the use of a piezoelectric force sensor. It is the easiest method of recording the distribution of hydraulic impulses generated in the water jets produced in the self-excited pulse head over a specific, generally very short period. Such tests were carried out at nominal pressures: 10, 15, 20, and 25 MPa at a distance of the head from the force sensor equal to 50 mm.

#### 6.4.1. The Frequency of Occurrence of Impulses in the Water Stream in the Air Environment

**Figure 20:**
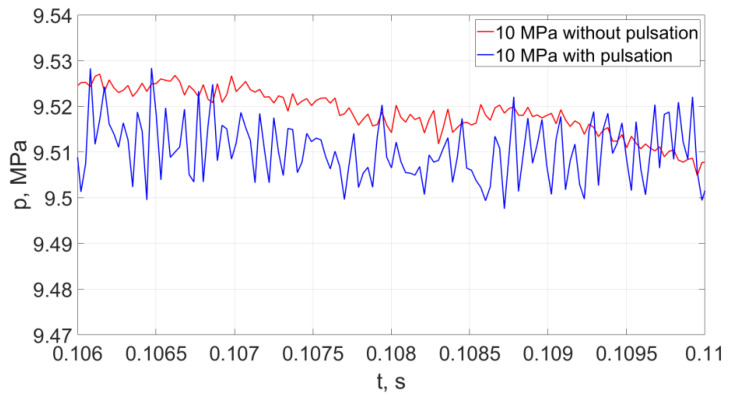
Distributions of hydrodynamic impulses occurring in the water stream (with a pressure of 10 MPa at a distance of 50 mm from the outlet from the pulsating head), produced in the head for drilling holes in the air environment and in the “continuous” stream operating under similar conditions.

The frequency of occurrence of hydraulic pulses in the water stream generated in the air-environment drilling head was tested at various nominal pressures (10, 15, 20, 25 MPa). A piezoelectric force sensor of the KISTLER 9602AQ01 type was used in the measurements, which was placed at a distance of 50 mm from the outlet from the pulse head. Examples of the results of the distribution of hydrodynamic pulses and the waveforms of the water jet thrust, selected for the same time interval of 4 ms, which were recorded in water jets operating in the air environment at different nominal pressures, are shown in Figure 20.

The analysis of the distribution of hydraulic impulses showed that with each increase in nominal water pressure by 5 MPa, the pressure of such impulses increases. For the lowest pressures of the water stream, such a temporary increase in pressure is about 53% and successively: for average values of the pressure of the stream, it increases by about 35%, and the smallest increase (about 26%) occurs for the water stream generated at the highest nominal pressures.

Variable values of the thrust force generated in the self-excited pulsation head intended for surface treatment, recorded at nominal pressures of 10 ÷ 25 MPa, confirm the occurrence of hydraulic impulses. With the increase of the nominal water pressure, the frequency of the impulse of the thrust forces decreases. The recorded course of the water jet thrust force with each increase in pressure by 5 MPa is characterized by a temporary increase in the thrust force by about 15.8 N for the lowest and average pressures and by about 11 N for the highest pressures (in the range of 15–20 MPa).

**Figure 21:**
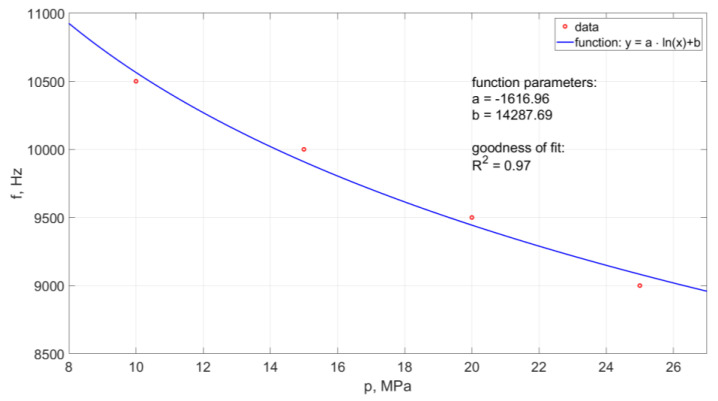
Influence of nominal water pressure on the pulsation frequency of the water stream generated in the head designed for drilling holes in the air environment.

Moreover, the frequency of pulsation formation in the water stream decreased with the increase of the nominal water pressure. A graphic illustration of the course of changes in the pulsation frequency of the water stream is shown in Figure 21.

The course of changes in the frequency of pulsations depending on the nominal water pressure is described by the following empirical formula:

(5) f=−1616.96·ln(p)+14287.69

The model determination coefficient is approximately R^2^ = 0.97. Thus, in approximately 97% the above model explains the variability of f(p).

#### 6.4.2. The Frequency of Pulses in the Water Stream Operating in the Water Environment

The frequency of occurrence of hydraulic pulses in the water stream generated in the drilling head was also tested in the water environment. Measurements were made by installing a piezoelectric force sensor in a water reservoir at a distance of 50 mm from the pulse head.

**Figure 22:**
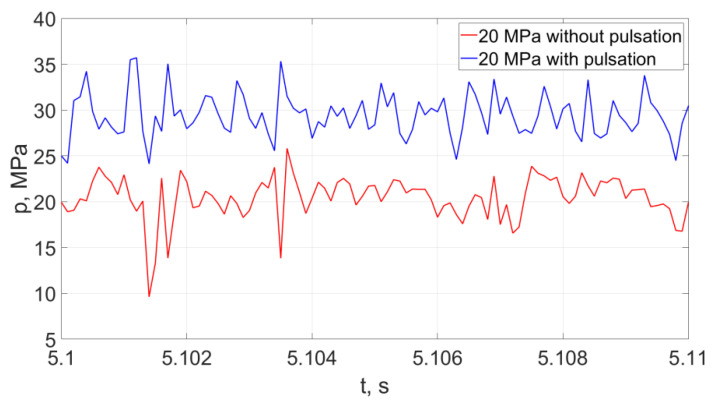
Distributions of hydrodynamic impulses occurring in the water stream (with a pressure of 20 MPa at a distance of 50 mm from the outlet from the pulsating head), produced in the head for drilling holes in the water environment and in the “continuous” stream operating under similar conditions.

Representative examples of instantaneous pressure distributions and thrust forces of pulsating and continuous water jets, recorded with a force sensor with a KISTLER 9602AQ01 piezoelectric transducer during 10 ms, at different nominal pressures (20, 25 MPa) are presented in Figure 22.

The analysis of the distribution of hydrodynamic impulses shows that with an increase in nominal water pressure by 5 MPa, there is a clear increase in the temporary pressure of hydrodynamic impulses. For the lowest pressures of the water stream, the temporary pressure may increase by 100%. For average nominal pressure values (in the order of 15 ÷ 20 MPa), the temporary pressure increases to 50%, while for the water stream generated at the highest nominal pressures, the average values of increasing the temporary pressures of hydrodynamic impulses are 17%.

The courses of the water jet thrust forces with each increase in pressure by 5 MPa indicate a temporary increase in the thrust force by about 12.6 N for the lowest pressures and about 9.1 N for the average and highest pressures (in the range of 15–20MPa and 20–25 MPa).

**Figure 23:**
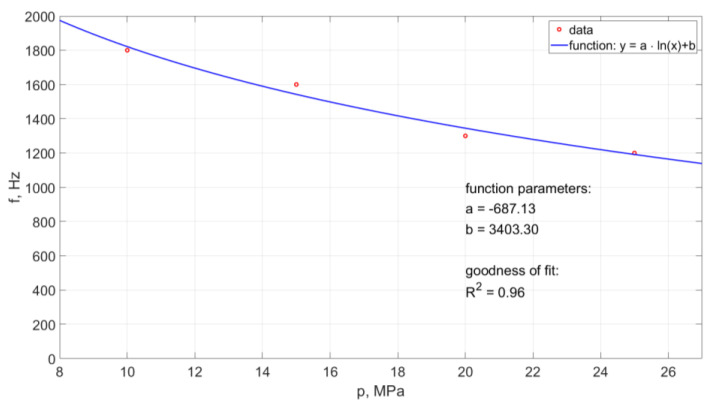
The waveform of the frequency of hydrodynamic pulses recorded with a force sensor for a self-excited pulse head designed for drilling holes working in an aquatic environment.

As a result of the research, it was also found that the frequency of pressure pulsation in the water stream decreases with the increase of nominal water pressure. An example of the pulsation frequency diagram of such a water stream is shown in Figure 23.

Such a course of the frequency of occurrence of hydrodynamic pulses can be described by the following empirical relationship:

(6) f=−687.13·ln(p)+3403.3

Based on this relationship (with the accuracy of matching defined by a high regression coefficient R^2^ = 0.96), it is possible to determine the frequency of occurrence of hydrodynamic pulses at different pressures within the studied range of their variability.

### 6.5. Erosiveness of the Pulsating Water Stream

The assessment of the erosiveness of the pulsed stream is one of the criteria determining the suitability of the head for drilling holes. Water jet erosivity tests consist in assessing the depth of the groove etched in the processed material during a single passage of the head 50 mm away from it with a selected feed speed.

Due to the technological possibilities of constructing a laboratory stand to carry out tests of the erosiveness of a pulsed stream in an aqueous environment, in which there would be no uncontrolled “splashes” of the stream in laboratory conditions, it was possible to conduct only such erosivity tests for the head working in the air environment.

**Figure 24:**
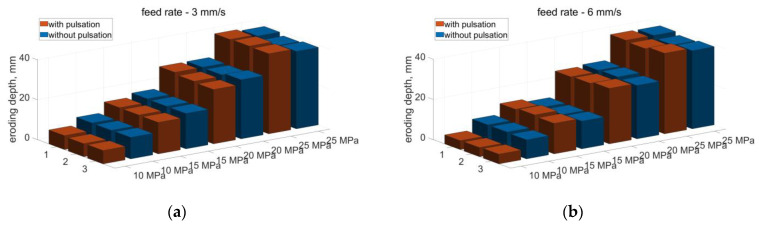
Comparison of the eroding depth of aerated concrete with a water jet (pulsating and without pulsation) produced in the drilling head with a feed rate of: (**a**) 3 mm/s, (**b**) 6 mm/s.

A summary of the measured depths of pulsed eroding and continuous water jets produced in the head operating in the air environment is shown in Figure 24 (for the feed speed of 3 and 6 mm/s).

Based on the obtained erosion results of the material processed with the water jet produced in the head intended for drilling holes, working in the air environment, it appears that for the tested ranges of pressure and feed speed, no significant increase in the erosivity of the pulsating water jet was found in relation to the jet without pulsation.

## 7. Conclusions

The analysis of the results of computer simulation tests, illustrating the influence of individual dimensional parameters on the formation of compact vortex rings, and the achievement of the maximum speed of the water stream along with the suction of the external medium through the side holes of the self-excited pulsation head, made it possible to formulate the following conclusions:

- The distributions of velocity and pressure vectors as well as the shape of the vortex rings inside the head chamber with side openings situated at an angle of 120° do not favorably influence the formation of hydraulic impulses.
- The pulse head with side openings tangent to the swirl chamber creates a variable shape of the swirl rings, which disturb the water pulsing mechanism.
- The pulse head with side openings perpendicular to the axis of the vortex chamber favors the formation of stable water pulses with the favorable shape of the vortex rings and the pressure distribution.

The analysis of the laboratory tests carried out to characterize the water jet produced in the self-excited pulsed head intended for drilling holes, allows the formulation of the following conclusions:

- Analysis of the structure of the pulsating stream at the outlet of the head allowed to determine the average distance of pulses formation for the tested values of nominal pressures.
- With the increase of the nominal pressure value, different distances of the formation of water pulses were observed both for the head working in the air environment and in the water environment.
- The average distance of hydrodynamic impulses from the outlet of the head operating in the water environment is greater by 18 ÷ 25% than during operation in the air environment (in the range of the analyzed pressure values).
- Due to recorded images of head operation for nominal water pressures: 10, 15, 20, 25 MPa, in the air environment only the presence of water stream discontinuities were observed whereas in the water environment interrupted flows were observed
- An increase in the nominal water pressure causes a decrease in the frequency of hydrodynamic pulses, both in the water and air environment.
- With the increase of the nominal water pressure, the average values of the frequency of occurrence of hydrodynamic pulses generated in the self-excited pulse head, show a decrease in frequency with increasing water pressure.
- The highest average value of the frequency of occurrence of hydrodynamic impulses (10,417 Hz) was obtained for the head operating in the air environment at the nominal pressure of 10 MPa, which is about 570% of the increase compared to the head operating in the water environment, for which registered frequency at the same pressure was 1800 Hz.
- An increase in the nominal water pressure supplying the self-excited pulsating head intended for drilling holes causes an increase in the average value of the thrust force of the pulsating water stream operating both in the air and water environment.
- Self-excited pulsation head working in the water environment produces a water stream characterized by higher average values of the thrust force compared to the analogous water stream operating in the air environment. The largest, 50% increase in the thrust force occurs at the nominal water pressure of 20 MPa, while the smallest increase, reaching only 5%, was observed at the pressure of 10 MPa.
- Self-excited pulse head working in the water environment provides a significant increase in the dynamic pressure of the water stream, compared to the head working in the air environment. For medium and highest pressures, such an increase is almost 82%, while for the lowest pressures (10 MPa), the thrust values increase by 46%.
- The results of the erosion of the material processed with the water jet produced in the head intended for drilling holes, working in the air environment, showed that for the tested ranges of the nominal water pressure and the speed of the water jet, there was no significant increase in the erosivity of the pulsating water jet, compared to the analogous water jet without pulsation.

## Figures and Tables

**Table 1:** Ranges of the variability of the investigated geometric parameters for various design solutions of a self-excited pulse head operating at a pressure of 150 MPa.

Geometric Parameters	Range	Unit
inlet nozzle diameter, d_1_	2.5	mm
inlet nozzle height, h_1_	6; 8;10;12	mm
rake angle of the chamber, λ	100;110;120	°
side inlets diameter, d_2_	2;3;4;5;6;7;8	mm
swirl chamber diameter, D	20;25;30;35;40;45;50	mm
vortex chamber height, H	20;25;30;35;40;45;50	mm
outlet nozzle diameter, d_3_	4;5;6;7;8;9	mm

**Table 2:** Characteristics of the geometric dimensions and velocity of the water stream produced in the most favorable models of heads with side openings perpendicular to the swirl chamber.

		Models of Heads with Side Openings Perpendicular to the Swirl Chamber		
Parameter	Designation	(a)	(b)	(c)
inlet nozzle diameter, mm	d_1_	2.5	2.5	2.5
inlet nozzle height, mm	h_1_	12	12	10
rake angle of the chamber, °	λ	120	110	120
side inlets diameter, mm	d_2_	2	6	2
swirl chamber diameter, mm	D	25	32	30
swirl chamber height, mm	H	15	25	20
outlet nozzle diameter, mm	d_3_	4	7	4
average speed at outlet, m/s	υ_avg_.	264	224	242
maximum speed at inlet, m/s	υ_max_	590	556	561
speed ratio, %	υ_śr_/υ_max_	44.7	40.3	43.1

**Table 3:** Characteristics of the geometric dimensions and velocity of the water stream produced in the heads optimal in terms of the location of the side holes.

Parameter	Designation	Side Openings at an Angle of 120° to the Head Axis	Side Openings Tangent to the Swirl Chamber	Side Openings Perpendicular to the Swirl Chamber
swirl chamber diameter, mm	D	25	30	25
swirl chamber height, mm	H	30	30	15
rake angle of the swirl chamber, °	λ	100	120	120
inlet nozzle height, mm	h_1_	8	6	12
inlet nozzle diameter, mm	d_1_	2.5	2.5	2.5
side inlets diameter, mm	d_2_	3	2	2
outlet nozzle diameter, mm	d_3_	6	4	4
average speed at outlet, m/s	υ_avg_.	113	279	264
maximum speed at inlet, m/s	υ_max_	561	589	590
speed ratio, %	υ_śr_/υ_max_	20.1	47.4	44.7

**Table 4:** Geometric parameters of the self-excited pulse head taken into account during the optimization process of the selected solution.

Lp	Parameter	Designation	Range
1	width of the annular surfaces of the swirl chamber, mm	SP	1 ÷ 6
2	outlet nozzle height, mm	h_3_	4 ÷ 24
3	swirl chamber diameter, mm	D	25 ÷ 50
4	vortex chamber height, mm	H	15 ÷ 35
5	inlet nozzle height, mm	h_1_	6 ÷ 12
6	rake angle of the swirl chamber, °	λ	100 ÷ 120
7	outlet nozzle diameter, mm	d_3_	4 ÷ 9
8	side holes diameter, mm	d_2_	2 ÷ 5
9	inlet nozzle diameter, mm	d_1_	2 ÷ 3.2

**Table 5:** Selected dimensional parameters of the selected model of a self-excited pulsation head intended for drilling holes with a water jet for pressures from 15 MPa to 150 MPa.

Lp	Parameter	Designation	Value
1	swirl chamber diameter, mm	D	25
2	swirl chamber height, mm	H	15
3	inlet nozzle height, mm	h_1_	12
4	rake angle of the swirl chamber, °	λ	120
5	outlet nozzle diameter, mm	d_3_	4
6	side holes diameter, mm	d_2_	2
7	inlet nozzle diameter, mm	d_1_	2.5
8	width of the annular surfaces of the swirl chamber, mm	SP	6

**Table 6:** Selected dimensional parameters of the selected model of a self-excited pulsation head intended for drilling holes with a water jet for pressures from 15 MPa to 150 MPa.

Lp.	Pressure P, MPa	Pulse Formation Distance l_i,_ mm					The Average Distance of the Formation of the Pulses, mm
1	10	24.44	25.44	24.96	25.45	25.1	25.08
2	15	35.85	35.22	35.81	35.82	35.72	35.68
3	20	41.97	41.23	41.51	40.98	41.02	41.34
4	25	41.57	42.51	42.33	41.52	42.58	42.10

**Table 7:** Measurements of the distance between the shaping of hydrodynamic impulses in the water stream flowing from the outlet nozzle of the head intended for drilling holes, working in the water environment.

lp.	Pressure p, MPa	Pulse Formation Distance l_i_, mm					The Average Distance of the Formation of the Pulses, mm
1	10	30.7	31.1	30.8	30.2	30.1	30.58
2	15	46.5	46.2	47.1	46.8	46.9	46.70
3	20	50.3	50.8	50.2	51.3	50.9	50.70
4	25	55.8	56	56.2	55.4	55.6	55.80

## Data Availability

Data sharing is not applicable to this article.
